# Anticancer Activity of (±)-Kusunokinin Derivatives towards Cholangiocarcinoma Cells

**DOI:** 10.3390/molecules27238291

**Published:** 2022-11-28

**Authors:** Thidarath Rattanaburee, Patpanat Sermmai, Kornthip Tangthana-umrung, Tienthong Thongpanchang, Potchanapond Graidist

**Affiliations:** 1 Department of Biomedical Sciences and Biomedical Engineering, Faculty of Medicine, Prince of Songkla University, Hat Yai 90110, Songkhla, Thailand; 2 Department of Chemistry and Center of Excellence for Innovation in Chemistry (PERCH-CIC), Faculty of Science, Mahidol University, Bangkok 10400, Thailand; 3 The Excellent Research Laboratory of Cancer Molecular Biology, Prince of Songkla University, Songkhla 90110, Thailand

**Keywords:** anticancer, cell arrest, apoptosis, (±)-kusunokinin, derivative

## Abstract

This study aimed to investigate the cytotoxicity and anticancer activity of (±)-kusunokinin derivatives ((±)-TTPG-A and (±)-TTPG-B). The cytotoxicity effect was performed on human cancer cells, including breast cancer, cholangiocarcinoma, colon and ovarian cancer-cells, compared with normal cells, using the MTT assay. Cell-cycle arrest and apoptosis were detected using flow-cytometry analysis. We found that (±)-TTPG-B exhibited the strongest cytotoxicity on aggressive breast-cancer (MDA-MB-468 and MDA-MB-231) and cholangiocarcinoma (KKU-M213), with an IC50 value of 0.43 ± 0.01, 1.83 ± 0.04 and 0.01 ± 0.001 µM, respectively. Interestingly, (±)-TTPG-A and (±)-TTPG-B exhibited less toxicity than (±)-kusunokinin (9.75 ± 0.39 µM) on L-929 cells (normal fibroblasts). Moreover, (±)-TTPG-A predominated the ell-cycle arrest at the S phase, while (±)-TTPG-B caused cell arrest at the G0/G1 phase, in the same way as (±)-kusunokinin in KKU-M213 cells. Both (±)-TTPG-A and (±)-TTPG-B induced apoptosis and multi-caspase activity more than (±)-kusunokinin. Taken together, we conclude that (±)-TTPG-A and (±)-TTPG-B have a strong anticancer effect on cholangiocarcinoma. Moreover, (±)-TTPG-B could be a potential candidate compound for breast cancer and cholangiocarcinoma in the future.

## 1. Introduction

Cancer is the leading cause of death worldwide. In 2020, new cases of all cancers were 19.3 million, and cases of death were 10.0 million [1]. In Thailand, the top five new cancers are liver, lung, breast, colorectum and cervix uteri, in both sexes [1]. Cholangiocarcinoma (CCA) is a malignant tumor originating from the bile-duct epithelial cells [2]. Globally, CCA approximates to around 10% to 20% of primary liver cancers. The global mortality of CCA patients has increased over many years. The highest incidence of global mortality for CCA was reported in northeastern Thailand [3]. CCA carries a very poor prognosis, due to usually being asymptomatic in the early stages [2]. Therefore, CCA is often diagnosed at advanced stages [4]. General cancer-treatments are surgery, radiation therapy, hormonal therapy and chemotherapy [5]. The type of chemotherapy depends on the tumor type and the sensitivity of the tumor [6]. For example, the combination of gemcitabine and cisplatin remains the current first-line chemotherapy regimen for patients with advanced CCA [2]. Chemotherapeutic drugs for breast cancer are doxorubicin, epirubicin, 5-fluorouracil (5-FU), tamoxifen, docetaxel, herceptin, paclitaxel, methotrexate, cyclophosphamide and carboplatin [7,8]. Commonly used medications for colorectal cancer such as 5-fluorouracil (5-FU) and oxaliplatin are administered in the adjuvant setting [9]. The National Comprehensive Cancer Network (NCCN) recommend the use of gemcitabine, capecitabine, or 5-fluorouracil (5-FU) for advanced biliary-tract cancer [10,11]. Cisplatin and paclitaxel are the two standard chemotherapeutic drugs for ovarian cancer [12]. Unfortunately, chemotherapeutic drugs cause adverse effects including nausea, vomiting, hair loss, cognitive dysfunction, fatigue, changes in sexual functioning, and reductions in quality-of-life ratings [13]. Anti-cancer agents from natural sources are categorized by a chemical structure including alkaloids, flavonoid, terpenoid, taxanes and lignan [14]. Chemotherapeutic drugs in an alkaloid compound are actinomycin D, dactinomycin, daunorubicin, doxorubicin, epirubicin, idarubicin, mitomycin, vinblastine, vindesine, vincristine and vinorelbine [15]. Flavonoid compounds are silymarin, genistein, quercetin, daidzein, luteolin, kaempferol, apigenin and epigallocatechin 3-gallate [16]. Potential chemotherapeutic agents in the terpenoid group include geraniol, andrographolide, excisanin A, gnidimacrin, oridonin and actein [17]. Chemotherapy agents in the taxane compound are paclitaxel and docetaxel [18]. Unfortunately, most patients develop resistance to chemotherapy drugs when used for a long time.

The compound (−)-Kusunokinin ((3R,4R)-3-(1, 3-benzodioxol-5-ylmethyl)-4-[(3, 4-dimethoxyphenyl) methyl] oxolan-2-one), a lignan compound from Piper nigrum, exhibits cytotoxicity in breast and colorectal cancer-cells. This compound induces cell-cycle blockage, apoptosis and cell-cycle arrest at the Gap 2/mitotic (G2/M) phase in breast-cancer cells (MCF-7) [19]. In addition, synthetic (±)-kusunokinin also exhibits cytotoxic activity in many cancer cells including breast, cholangiocarcinoma, colon and ovarian cancer-cells. This synthetic compound inhibits cell proliferation through the suppression of topoisomerase II, STAT3, CyclinD1 and p21, and also induces apoptosis through the increase of multi-caspase activity [20]. Natural (−)-kusunokinin inhibits tumor growth and tumor-related protein in breast-cancer rats [21]. For the target proteins of (−)-kusunokinin, we reported that (−)-Kusunokinin bound CSF1R, which consequently affected AKT and protein-associated cell proliferation, including CyclinD1 and CDK [22]. Moreover, (−)-kusunokinin also bound AKR1B1, the upstream molecules of the AKT signaling-protein [23]. The chemical structure of (−)-kusunokinin consists of two active parts (3,4 di-methoxybenzyl butyrolactone and 1,3-benzodioxole) which bind CSF1R and AKR1B1 with a non-covalent bond. Therefore, the binding of (−)-kusunokinin with target proteins still shows the less specific, rather than known, inhibitors. For cytotoxic activity, synthetic (±)-kusunokinin represented less cytotoxicity in aggressive breast-cancer (MDA-MB-468 and MDA-MB-231) cells, when compared with low-aggressive breast cells (MCF-7). Furthermore, (±)-kusunokinin represented a stronger cytotoxicity effect against undifferentiated cholangiocarcinoma (KKU-M213) cells than against well-differentiated cholangiocarcinoma (KKU-K100 and KKU-M055) cells [20].

**Figure 1:**
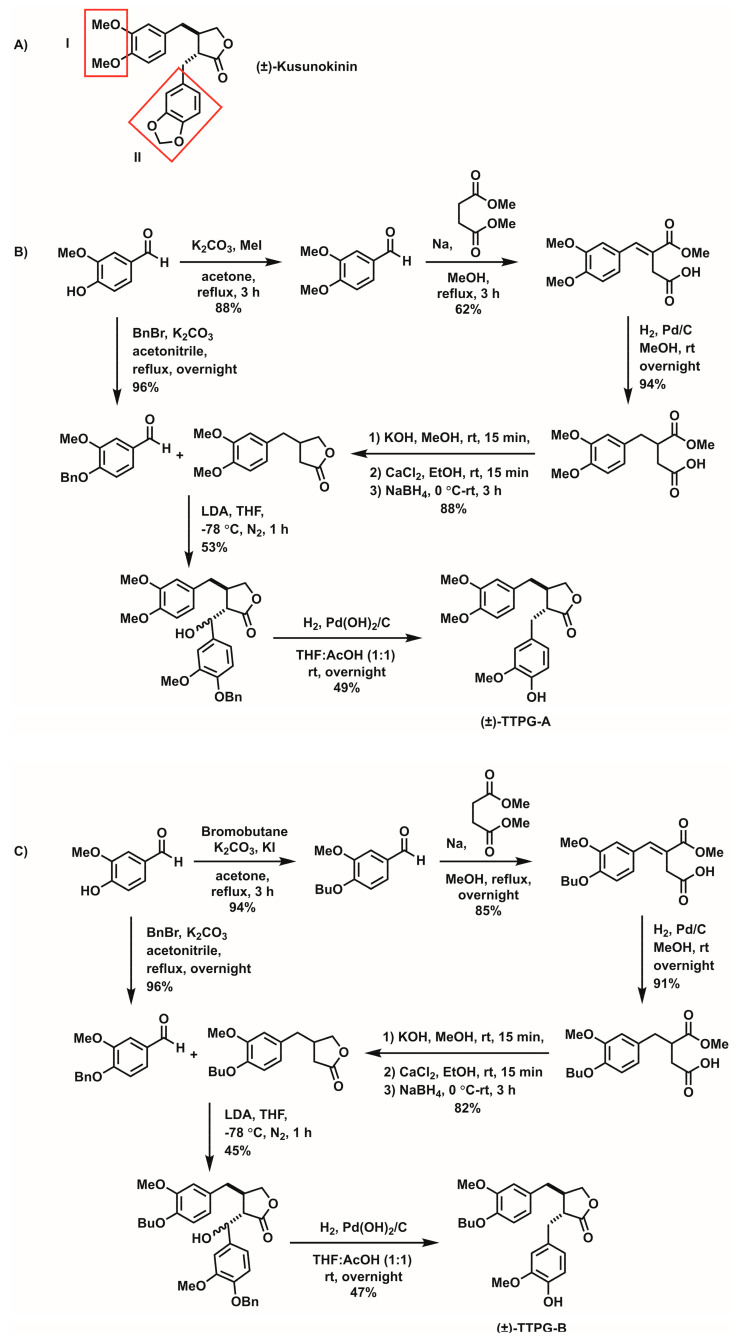
The chemical structure of (**A**) (±)-kusunokinin and the synthetic pathway of (**B**) (±)-TTPG-A and (**C**) (±)-TTPG-B. The chemical structures of (±)-TTPG-A and (±)-TTPG-B were modified at (I) the 3,4 dimethoxybenzyl butyrolactone part and (II) the 1,3-benzodioxole parts.

Due to the low cytotoxic-effect of (±)-kusunokinin in aggressive cancer-cells and low binding affinity oi target proteins, modification to the chemical structure of (±)-kusunokinin was necessary. In this study, (±)-kusunokinin was modified to generate derivative compounds at two specific parts (3,4 dimethoxybenzyl butyrolactone and 1,3-benzodioxole) (Figure 1). We investigated the anti-cancer effect of (±)-kusunokinin de-rivatives ((±)-TTPG-A and (±)-TTPG-B)) on cancer cells, and determined the inhibition effect on cell-cycle arrest, multi-caspase activity and apoptosis.

## 2. Results

### 2.1. The Cytotoxic Effect of (±)-TTPG-A and (±)-TTPG-B on Four Types of Cancer and Normal Cells

Cytotoxicity experiments were performed on four cancerous cells, along with three normal cells, using an MTT assay. The cytotoxicity of (±)-TTPG-A and (±)-TTPG-B (IC_50_ values) is shown in Table 1. Results showed that (±)-TTPG-B exhibited the strongest cytotoxicity in aggressive-breast-cancer cells (MDA-MB-468 (0.43 ± 0.01 µM) and MDA-MB-231 (1.83 ± 0.04 µM)). In addition, the highest IC_50_ values of (±)-TTPG-A and (±)-TTPG-B were 0.07 ± 0.01 µM and 0.01 ± 0.001 µM, respectively, in cholangiocarcinoma cells (KKU-M213), which were stronger than (±)-kusunokinin (4.47 ± 0.04 µM). For ovarian-cancer cells, (±)-TTPG-B represented stronger cytotoxicity than (±)-TTPG-A and (±)-kusunokinin (4.52 ± 0.03 µM). In addition, (±)-kusunokinin showed less cytotoxicity than (±)-TTPG-A and (±)-TTPG-B in MMNK-1 and Vero cells. Interestingly, neither compound, (±)-TTPG-A and (±)-TTPG-B, inhibited L-929 cells, while (±)-kusunokinin inhibited these cells with an IC_50_ value of 9.75 ± 0.39 µM. Due to the high cytotoxicity of both these compounds, KKU-M213 cells were selected for the following experiments.

### 2.2. (±)-TTPG-A and (±)-TTPG-B Exerted Cell-Cycle Arrest

**Figure 2:**
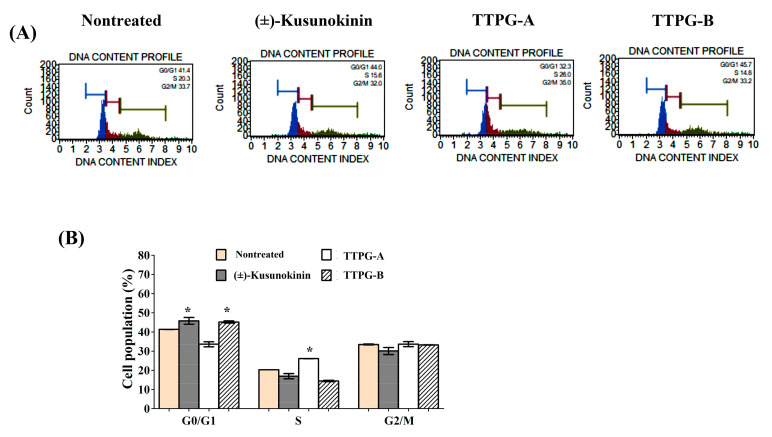
Induction of cell-cycle arrest by (±)-TTPG-A and (±)-TTPG-B on KKU-M213 cells. (**A**) Cells were treated with (±)-kusunokinin, (±)-TTPG-A and (±)-TTPG-B, at 4.47, 0.07 and 0.01 µM respectively, for 48 h, while untreated cells served as control. Cell-cycle distribution was measured using flow cytometry after staining with PI. DNA histograms display cell-cycle phase of treated cells, namely G0/G1, S, and G2/M (*n* = 3). The G0 phase is a resting phase, where cells leave the cycle and stop dividing. The G1 phase is the first phase within the interphase. The S phase starts when DNA synthesis commences, and completes when chromosomes have been replicated. The G2 phase is a period of protein synthesis and rapid cell-growth to prepare the cell for mitosis. The M phase is called the chromosome separation phase. (**B**) The proportion of cells according to the cell-cycle phase are presented by percentage mean ± SD (*n* = 3). * Indicates *p* < 0.05 concerning control.

The activity of (±)-TTPG-A and (±)-TTPG-B on cell-cycle distribution was evaluated through flow cytometry. PI staining was used to perform cell-cycle analysis. The results are summarized in Figure 2: (±)-Kusunokinin, (±)-TTPG-A and (±)-TTPG-B at 4.47, 0.07 and 0.01 µM were treated on KKU-M213 cells for 48 h. Untreated cells served as control. As seen from the results, (±)-TTPG-A cells arrest in the S phase (26.12 ± 0.11%), compared with the control (20.32 ± 0.001%). Furthermore, (±)-kusunokinin (16.94 ± 1.95%) and (±)-TTPG-A (14.40 ± 0.50%) decreased population in the S phase after 48 h incubation. In addition, (±)-kusunokinin and (±)-TTPG-A dominated the population of cells accumulating in the G0/G1 phase, compared with the control (41.34 ± 0.03%). These data indicate that (±)-TTPG-A and (±)-TTPG-B induced cell-cycle arrest at S and G0/G1, respectively.

### 2.3. (±)-TTPG-A and (±)-TTPG-B Induced Apoptotic Cells

**Figure 3:**
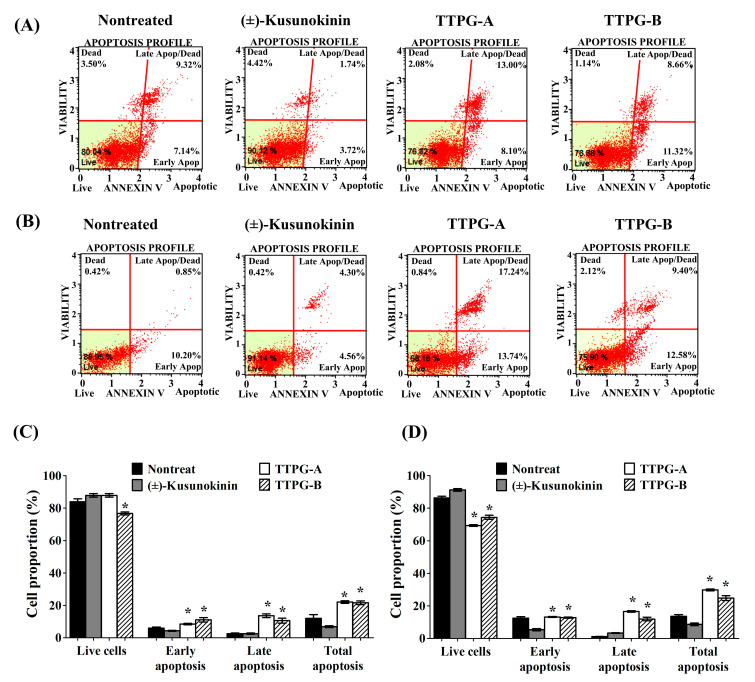
(±)-TTPG-A and (±)-TTPG-B induced apoptosis. (**A**) KKU-M213 cells were incubated with (±)-Kusunokinin, (±)-TTPG-A and (±)-TTPG-B at 4.47, 0.07 and 0.01 µM, respectively, for 72 h, and nontreated cells served as control. (**B**) Cells were treated with (±)-kusunokinin, (±)-TTPG-A and (±)-TTPG-B at 0.15 µM, and incubated for 48 h. After treatment, apoptotic cells were analyzed using the Muse^®®^ Annexin V-FITC assay and Propidium Iodide (dead-cell kit). The results of this experiment were analyzed using flow cytometry. Each plot has four quadrant markers. The lower-left and lower-right represent live and early-apoptotic cells, respectively. The upper-left and upper-right quadrants are dead cells (necrosis) and late-apoptotic/dead cells, respectively. (**C**,**D**) The graph represents the summary of average percentages ± SD of live, early-apoptotic, late-apoptotic and total-apoptotic cells from three independent experiments. The statistical analysis of the data was tested using Student’s *t*-test. * Indicates *p* values less than 0.05 which were considered as significant differences, compared with the control group.

To identify whether (±)-TTPG-A and (±)-TTPG-B inhibited cell-proliferation through apoptosis, this experiment performed an Annexin V/7-AAD apoptosis-detection assay (Figure 3). Treated cells were stained with Annexin V-FITC and PI (Propidium iodide), which can assess the early-apoptotic and late-apoptotic cell proportions. Due to the cytotoxicity effect, the IC_50_ value of (±)-kusunokinin (4.47 µM), (±)-TTPG-A (0.07 µM) and (±)-TTPG-B (0.01 µM) were chosen to test whether apoptotic cells occurred after treatment on KKU-M213 cells for 72 h. The percentage of early apoptosis of (±)-TTPG-A and (±)-TTPG-B represented around 9.10 ± 3.25% and 8.00 ± 0.85%, which was higher than (±)-kusunokinin (4.75 ± 1.42%) and the nontreated cells (4.74 ± 0.52%). Late apoptosis and total apoptosis were significantly raised after (±)-TTPG-A- and (±)-TTPG-B-treatment, compared with (±)-kusunokinin and non-treated cells (Figure 3A,C). Interestingly, treatment with 0.15 µM of (±)-TTPG-A and (±)-TTPG-B for 48 h resulted in significantly decreased live-cells when compared with (±)-kusunokinin and non-treated cells. Both (±)-TTPG-A and (±)-TTPG-B significantly increased the late apoptosis and total apoptosis, more than (±)-kusunokinin and nontreated cells (Figure 3B,D). These results indicate that (±)-TTPG-A and (±)-TTPG-B increase cytotoxicity via the apoptosis pathway.

### 2.4. (±)-TTPG-A and (±)-TTPG-B Increased Multi-Caspase Activity

**Figure 4:**
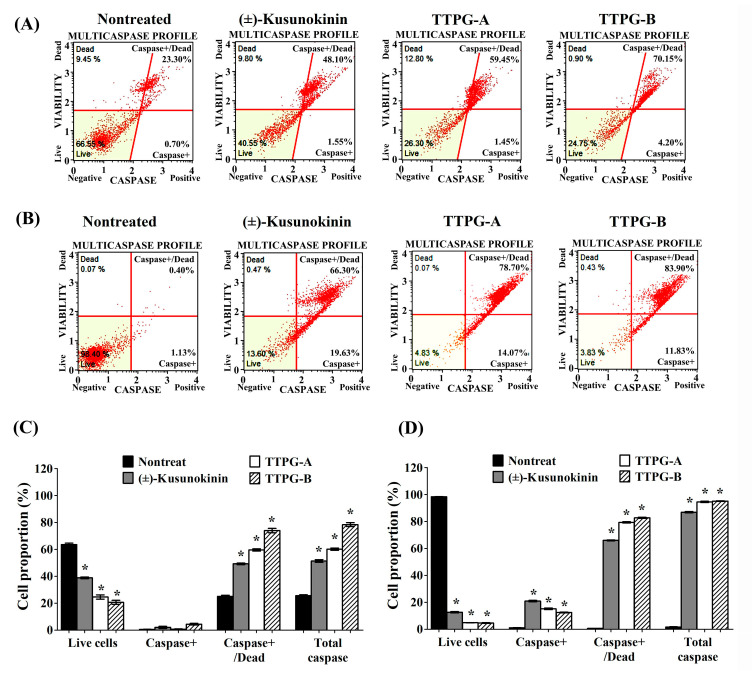
(±)-TTPG-A and (±)-TTPG-B enhanced the multi-caspase activity. (**A**) The concentrations of (±)-kusunokinin, (±)-TTPG-A and (±)-TTPG-B used in this treatment were 4.47, 0.07 and 0.01 µM, respectively, for 72 h. Non-treated cells served as control. (**B**) KKU-M213 cells were exposed to 0.15 µM of (±)-kusunokinin, (±)-TTPG-A and (±)-TTPG-B for 48 h. After treatment, cells were analyzed for the activities of multiple caspases (caspase-1, -3, -4, -5, -6, -7, -8, and -9), using the Muse^®®^ Multi Caspase assay and 7-AAD (dead-cell kit). Fluorescence intensity for multi-caspase was indicated on the x-axis, and 7-AAD was indicated on the y-axis. The populations of stained/unstained cells were indicated in each quadrant. Four populations of cells were distinguished for live cells (lower left for caspase(−) and 7-AAD(−)), caspase activity (lower right for caspase(+) and 7-AAD(−)), late stage of caspase activity (upper right for caspase(+) and 7-AAD(+)) and necrotic cells (upper left for caspase(−) and 7-AAD(+)). (**C**,**D**) The graph represents the average percentages ± SD of live, caspase+, caspase+/dead and total caspase from three independent experiments. The statistical analysis of the data was evaluated using Student’s *t*-test. * Indicates *p* values less than 0.05 which were considered as significant differences, compared with the control group.

During apoptosis, caspase activity contributes to the degradation of DNA, resulting in the modification of cell morphology and causing cell death. To investigate the induction of muti-caspases activity by (±)-TTPG-A and (±)-TTPG-B, KKU-M213 were exposed to each compound at IC_50_ concentration for 72 h (Figure 4A). Results showed that the live cells were significantly decreased after (±)-kusunokinin (37.95 ± 1.20%), (±)-TTPG-A (24.00 ± 3.08%) and (±)-TTPG-B (19.45 ± 2.95%) treatment, compared with the control cells (61.9 ± 2.22%). The percentage of caspase+/dead and total caspase of (±)-kusunokinin, (±)-TTPG-A and (±)-TTPG-B were significantly higher than in the non-treated cells (Figure 4C). After the treatment with 0.15 µM for 48 h, the live cells dramatically decreased in the (±)-kusunokinin, (±)-TTPG-A and (±)-TTPG-B treatments. The percentage of cell proportion of caspase+, caspase+/dead and total caspase of (±)-kusunokinin, (±)-TTPG-A and (±)-TTPG-B were significantly increased when compared with non-treated cells (Figure 4B,D). However, (±)-TTPG-A and (±)-TTPG-B increased caspase+/dead and total caspase more than (±)-kusunokinin (Figure 4C,D). These results indicate that (±)-TTPG-A and (±)-TTPG-B induced apoptosis through the induction of multi-caspase activity.

## 3. Discussion

In our previous study, we reported that natural (−)-kusunokinin showed anticancer activity against breast, colon and lung cancer [19,21]. In addition, the synthetic (±)-kusunokinin racemic-compounds also exhibited cytotoxic activity in many cancer cells including breast, cholangiocarcinoma, colon, and ovarian cancer cells [20,24]. In this present study, we aimed to evaluate the cytotoxicity of (±)-kusunokinin derivatives ((±)-TTPG-A and (±)-TTPG-B) in aggressive cancer-cells. The (±)-TTPG-B showed the strongest cytotoxicity in MDA-MB-468 and MDA-MB-231 cells which correspond to triple-negative breast cancer (TNBC), compared with (±)-kusunokinin and (±)-TTPG-A. Previously, we reported that synthetic (±)-bursehenin and (±)-kusunokinin derivatives showed IC_50_ values on MDA-MB-468 (8.24 ± 0.08 µM) [20], which were less effective than (±)-TTPG-A (6.38 ± 0.04 µM) and (±)-TTPG-B (0.43 ± 0.01 µM). Surprisingly, (±)-TTPG-A and (±)-TTPG-B exhibited the highest cytotoxicity in cholangiocarcinoma (KKU-M213) cells, which were stronger than (±)-kusunokinin. The synthetic (±)-bursehenin was effective against the KKU-M213 cells with IC_50_ values of 3.70 ± 0.79 µM [20], which is lower than (±)-TTPG-A and (±)-TTPG-B. There are many compounds which exhibit cytotoxicity against cholangiocarcinoma cells. For example, ursolic acid, a natural triterpenoid, has an IC_50_ value of 22.87 ± 1.77 µM on KKU-M213 cells. Cisplatin (a chemotherapeutic agent) has an IC_50_ value of (18.25 ± 8.30 µM) on KKU-M213 cells [25]. Moreover, lobaplatin, a platinum compound, inhibits cell proliferation in BRE cells (cholangiocarcinoma) with an IC_50_ value of 5.26 ± 0.63 µg/mL [26]. Atractylodin and β-eudesmol, from the rhizome of *Atractylodes lancea*, exhibit cytotoxic activity with IC_50_ values of 41.66 ± 2.51 µg/mL and 39.33 ± 1.15 µg/mL, respectively [27]. Telmisartan, an angiotensin receptor blocker, reduces cell proliferation in HuCCT-1 and TFK-1 cells [28]. β-eudesmol exhibits KKU-K100 cells with an IC_50_ value of 37.46±9.54 µM [29]. For the effect on ovarian cancer, (±)-TTPG-B had the highest cytotoxicity with the lowest IC_50_ value of 0.05 ± 0.01 µM. Many compounds show cytotoxicity on ovarian cancer. For example, deoxyschizandrin, a lignan compound, isolated from the fruits of *Schisandra chinensis*, shows a substantial growth-inhibitory effect on A2780 cells with an IC_50_ value of 27.81 ± 3.44 µM [30].

Surprisingly, (±)-TTPG-A and (±)-TTPG-B were less toxic than (±)-kusunokinin in L-929 (normal fibroblast) and HT-29 cells (colon cancer), which could be due to the specific targets of these two compounds. Etoposide is the most potent lignan-compound in chemotherapy [31]. Etoposide displays cytotoxicity against L-929 and MMNK-1 cells with IC_50_ values of 14.13 ± 0.39 µM and 5.51 ± 0.95 µM, respectively [21]. Taken together, the cytotoxicity of our (±)-kusunokinin derivatives especially (±)-TTPG-B, showed strong potential for aggressive breast cancer, cholangiocarcinoma and ovarian cancer and were less toxic in normal fibroblast cells.

**Figure 5:**
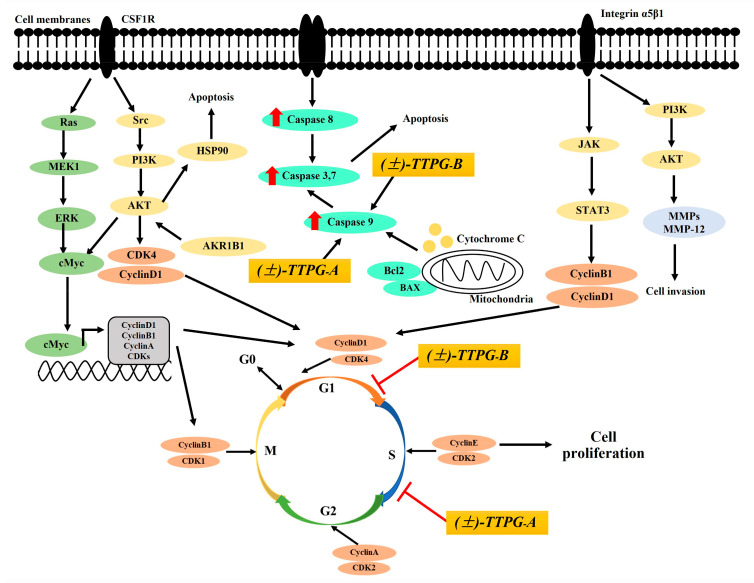
A schematic of the proposed anticancer activity of (±)-TTPG-A and (±)-TTPG-B on KKU-M213 cells. (±)-TTPG-A and (±)-TTPG-B increased protein in KKU-M213 cells, which are referred to by the red arrow. The red line represents cells-cycle arrest by (±)-TTPG-A and (±)-TTPG-B in KKU-M213 cells.

To understand the action of (±)-TTPG-A and (±)-TTPG-B on cell inhibition, we performed a cell-cycle-arrest assay. Results showed that (±)-TTPG-A and (±)-TTPG-B induced cell-cycle arrest at the S and G0/G1 phases, respectively. Cholangiocarcinoma cells (KKU-M213, KKU-K100 and KKU-M055) have high levels of integrin α5β1 [32]. This protein is involved in G1/S transition. FAK activates PI3K/AKT and MAPK/ERK signaling pathways, followed by the upregulation of CyclinD levels, and accelerates the degradation of the CDK inhibitors (p21, p27) [33]. The G1-phase progression was required for complexes of CDK4 and CDK6 with CyclinD1 (Figure 5) [34]. The beginning of the S phase is marked by increasing levels of Cyclin A, which binds CDK2. The complex formed by Cyclin A/CDK2 drives the cells through the S phase and promotes DNA replication [35]. In the present study, (±)-kusunokinin induced cell-cycle arrest at the G0/G1 phase on KKU-M213 cells. Previously, we reported that (−)-kusunokinin bound CSF1R and AKR1B1. (±)-Kusunokinin inhibited CSF1R and its downstream proteins, including AKT, CyclinD1 and CDK1. In addition, the possible target proteins of (−)-kusunokinin were MMP-12, HSP90α, CyclinB1, and MEK1 [22]. However, we indicated that (±)-kusunokinin inhibited CyclinD1 and CDK1 on KKU-M213 cells. (±)-TTPG-A and (±)-TTPG-B were (±)-kusunokinin-derivative compounds which modified at the binding position, including the 3,4 dimethoxybenzyl butyrolactone part and the 1,3-benzodioxole parts by adding butanol and hydroxyl groups. (±)-TTPG-A and (±)-TTPG-B increased cell-cycle arrest at the S and G0/G1 phase, respectively. The complex formed by Cyclin A/CDK2 and CyclinD1/CDK4 drives the cells through the S and G1 phase, respectively, [34,35]. Therefore, the proteins associated with the S and G0/G1 phase might be modulated by the (±)-TTPG-A and (±)-TTPG-B compound. A schematic picture of the anticancer activity of (±)-TTPG-A and (±)-TTPG-B is shown in Figure 5). Moreover, telmisartan induced G0/G1 cell-cycle arrest in HuCCT-1 cells [28]. Atractylodin and β-eudesmol also promoted cell-cycle arrest at the G1 phase on CL-6 and HUCC-T1 cells after 48 h of exposure [27]. Lobaplatin induced the accumulation of cells in the G0/G1 phase in BRE cells [26]. Cryptotanshinone caused RBE cells to arrest at the S phase, and downregulated the level of CyclinA1 [36]. ChromomycinA3, an anthraquinone glycoside-mithramycin A analog, inhibited cell-cycle progression at the S phase with a low dose in KKU-M213 cells [37]. Cancer progression and prognosis are correlated with the expression level of various cell-related molecules [35]. These data indicate that (±)-TTPG-A and (±)-TTPG-B may be activated as the major cell-cycle regulators of human cholangiocarcinoma cells.

In the apoptosis pathway, caspases are intracellular cysteine-protein enzymes that play a major role in the apoptotic mechanisms. Caspase-3/7 is the final molecule in the apoptosis pathway for both intrinsic and extrinsic pathways [38]. In this present study, (±)-TTPG-A and (±)-TTPG-B increased apoptotic cells via the modulation of multi-caspase activity on KKU-M213 cells. However, (±)-kusunokinin induced the apoptotic cells less than non-treated cells but (±)-kusunokinin showed significantly increased multi-caspase activity, more than non-treated cells. This phenomenon could be due to the dead cells being washed out during the cell harvest. Ursolic acid enhances apoptotic cells, along with also augmenting caspase-3/7 activity on KKU-M213 cells [25]. ChromomycinA3 promotes caspase-dependent apoptosis. Lobaplatin, atractylodin and β-eudesmol increase the level of caspase-3, which contributes to apoptosis [26]. Cryptotanshinone increases the amount of cleaved caspase-3 and cleaved caspase-9 in a dose-dependent manner in HCCC-9810 and RBE cells [36].

## 4. Materials and Methods

### 4.1. Synthesis of (±)-Kusunokinin, (±)-TTPG-A and (±)-TTPG-B Compound

#### 4.1.1. Synthesis of (±)-Kusunokinin

(±)-Kusunokinin was synthesized following the procedure reported by Ganeshpure and Stevenson [39].

#### 4.1.2. Synthesis (±)-TTPG-A

##### Preparation of 3,4-Dimethoxybenzaldehyde

To a suspension of vanillin (20.22 g, 132.89 mmol) and potassium carbonate (K_2_CO_3_) (45.92 g, 332.25 mmol) in 500 mL of acetone, was added methyl iodide (MeI) (12.4 mL, 199.31 mmol). The reaction mixture was then refluxed overnight. After this, the reaction mixture was cooled down and filtered, to remove the remaining K_2_CO_3_. Water (200 mL) was added to the filtrate and the mixture was extracted with ethyl acetate (3 × 200 mL). The organic layer was then combined, dried over Na_2_SO_4_, filtered, and evaporated to afford 3,4-dimethoxybenzaldehyde (19.45 g, 117.04 mmol, 88%) as yellow oil. The product was used in the next step without further purification. ^1^H NMR (400 MHz, CDCl_3_): *δ* 3.97 (*s*, 3H), 4.01 (*s*, 3H), 6.99 (*d*, *J* = 8.7 Hz, 1H), 7.40–7.51 (*m*, 2H), 9.85 (*s*, 1H); ^13^C NMR (100 MHz, CDCl_3_): *δ*55.8, 56.0, 108.8, 110.3, 126.7, 129.9, 149.4, 154.3, 190.6; HRMS (ESI+, *m*/*z*): calculated for C_9_H_10_O_3_ [M+Na]^+^ 189.0522, found [M+Na]^+^ 189.0533.

##### Preparation of 4-(3,4-Dimethoxyphenyl)-3-(methoxycarbonyl)but-3-enoic Acid

Under N_2_ atmosphere, Na metal (4.52 g, 196.69 mmol) was slowly added to 100 mL of dried methanol (MeOH), to generate sodium methoxide (NaOMe). After this, dimethyl succinate (7.03 mL, 53.78 mmol) was slowly added to the suspension of NaOMe, followed by stirring continuously at room temperature for 15 min. Then, the solution of 3,4-dimethoxybenzaldehyde (7.98 g, 48.00 mmol) in dry MeOH (50 mL) was added to the suspension, and then the mixture was refluxed for 3 h. After completion, the reaction mixture was cooled down and then worked up with conc. HCl until the pH of the solution was 1–2. Next, the reaction mixture was evaporated, to remove the MeOH. Then H_2_O (50 mL) was added, and the mixture was extracted with ethyl acetate (3 × 100 mL). The organic layer was then combined, dried over Na_2_SO_4_, and concentrated in vacuo. The crude product was purified using column chromatography with 30–50% EtOAc/hexane as an eluent to afford 4-(3,4-dimethoxyphenyl)-3-(methoxycarbonyl)but-3-enoic acid (8.34 g, 29.76 mmol, 62%) as a yellow solid. ^1^H NMR (400 MHz, CDCl_3_): *δ*3.63 (*s*, 2H), 3.86 (*s*, 3H), 3.91 (*s*, 6H), 6.91 (*d*, *J* = 8.8 Hz, 1H), 7.00–7.02 (*m*, 2H), 7.87 (*s*, 1H); ^13^C NMR (100 MHz, CDCl_3_): *δ*35.5, 52.3, 56.0, 56.1, 112.7, 113.3, 124.4, 125.9, 129.3, 140.16, 149.6, 150.2, 168.4, 170.0; HRMS (ESI+, *m*/*z*): calculated for C_14_H_16_O_6_ [M+Na]^+^ 303.0839, found [M+Na]^+^ 303.0822.

##### Preparation of 3-(3,4-Dimethoxybenzyl)-4-methoxy-4-oxobutanoic Acid

A suspension of 4-(3,4-dimethoxyphenyl)-3-(methoxycarbonyl)but-3-enoic acid (1.47 g, 5.23 mmol) and 10% Pd/C (0.026 g, 0.25 mmol) in MeOH (10 mL) was stirred at room temperature under a hydrogen (H_2_) atmosphere overnight. After this, the reaction mixture was filtered through a celite pad, and washed with MeOH twice. The filtrate was concentrated in vacuo to provide 3-(3,4-dimethoxybenzyl)-4-methoxy-4-oxobutanoic acid (1.39 g, 4.91 mmol, 94%) as a colorless oil. The product was used in the next step without further purification. ^1^H NMR (400 MHz, CDCl_3_): *δ*2.37–2.43 (*dd*, *J* = 17.2 Hz, 4.8 Hz, 1H), 2.62–2.69 (*m*, 2H), 2.92–3.07 (*m*, 2H), 3.62 (*s*, 3H), 3.81 (*d*, *J* = 3.3 Hz, 6H), 6.62 (*s*, 1H), 6.65 (*d*, *J* = 2.0 Hz, 1H), 6.74 (*d*, *J* = 7.9 Hz, 1H), 8.63 (*s*, 1H); ^13^C NMR (100 MHz, CDCl_3_): *δ*34.9, 37.3, 43.1, 52.1, 2 × 55.9, 111.3, 112.1, 121.2, 130.5, 147.9, 149.0, 174.8, 177.6; HRMS (ESI+, *m*/*z*): calculated for C_14_H_18_O_6_ [M+Na]^+^ 305.0996, found [M+Na]^+^ 305.0999.

##### Preparation of 4-(3,4-Dimethoxybenzyl)dihydrofuran-2(3H)-one

A solution of 3-(3,4-dimethoxybenzyl)-4-methoxy-4-oxobutanoic acid (1.58 g, 5.61 mmol) in MeOH 5 (mL) was stirred at room temperature in the presence of KOH (0.32 g, 5.61 mmol) for 15 min. After this, the reaction mixture was evaporated to dryness. Next, 20 mL of ethanol (EtOH) was added to the suspension, followed by CaCl_2_ (1.55 g, 13.97 mmol), and the mixture was stirred continuously for 15 min. Then, the mixture of KOH (0.13 g, 2.25 mmol) and NaBH_4_ (0.81 g, 21.28 mmol) in EtOH (5 mL) was slowly added to the reaction mixture at 0 °C, followed by stirring at room temperature for 3 h. After completion, the reaction mixture was quenched with conc. HCl until the pH reached 4–5, and then the solid was filtered out over a celite pad. The filtrate was extracted with EtOAc (3 × 50 mL). The resulting organic solutions were combined, dried over Na_2_SO_4_, filtered and concentrated in vacuo to generate 4-(3,4-dimethoxybenzyl) dihydrofuran-2(3H)-one in 88%, without any purification. ^1^H NMR (400 MHz, CDCl_3_): *δ*2.25–2.32 (*m*, 1H), 2.56–2.63 (*m*, 1H), 2.70–2.73 (*m*, 2H), 2.80–2.90 (*m*, 1H), 3.86 (*s*, 6H), 4.01–4.05 (*m*, 1H), 4.30–4.35 (*m*, 1H), 6.68–6.71 (*m*, 2H), 6.80–6.82 (*m*, 1H); ^13^C NMR (100 MHz, CDCl_3_): *δ*34.2, 37.3, 38.5, 55.9, 56.0, 72.7, 111.5, 111.9, 120.7, 131.0, 147.9, 149.1, 177.1; HRMS (ESI+, *m*/*z*): calculated for C_13_H_16_O_4_ [M+Na]^+^ 259.0941, found [M+Na]^+^ 259.0947.

##### Preparation of 4-(Benzyloxy)-3-methoxybenzaldehyde

To a suspension of vanillin (10.33 g, 67.89 mmol) and potassium carbonate (K_2_CO_3_) (9.47 g, 68.52 mmol) in acetonitrile (90 mL), was added benzyl bromide (BnBr) (10.1 mL, 85.03 mmol). The reaction mixture was then refluxed overnight. After this, the reaction mixture was cooled down and filtered to remove the remaining K_2_CO_3_. The filtrate was added to H_2_O (100 mL) and the mixture was extracted with ethyl acetate (3 × 100 mL). The organic layer was then combined, dried over Na_2_SO_4_, filtered and evaporated to dryness. The crude product was purified using column chromatography with 2:8 EtOAc:hexane as an eluent to afford 4-(benzyloxy)-3-methoxybenzaldehyde (15.75 g, 65.01 mmol, 96%) as a white solid. ^1^H NMR (400 MHz, CDCl_3_): *δ*3.95 (*s*, 3H), 5.25 (*s*, 2H), 6.99 (*d*, *J* = 8.2 Hz, 1H), 7.30–7.36 (*m*, 1H), 7.36–7.42 (*m*, 3H), 7.42–7.47 (*m*, 3H), 9.83 (*s*, 1H); ^13^C NMR (100 MHz, CDCl_3_): *δ*56.0, 70.9, 109.3, 112.4, 126.6, 2×127.2, 128.2, 2×128.7, 130.3, 136.0, 150.1, 153.6, 190.9; HRMS (ESI+, *m*/*z*): calculated for C_15_H_14_O_3_ [M+Na]^+^ 265.0835, found [M+Na]^+^ 265.0839.

##### Preparation of 3-((4-(Benzyloxy)-3-methoxyphenyl)(hydroxy)methyl)-4-(3,4-dimethoxybenzyl)dihydrofuran-2(3H)-one

Under a N_2_ atmosphere, 2M lithium diisopropylamide (LDA) (2.6 mL, 5.20 mmol) was slowly added to the mixture of 4-(3,4-dimethoxybenzyl)dihydrofuran-2(3H)-one (1.025 g, 4.34 mmol) in dry THF (17 mL) at −78 °C, and the reaction mixture was allowed to stir for 30 min. Then, 4-(benzyloxy)-3-methoxybenzaldehyde (1.06 g, 4.69 mmol) in dry THF (15 mL) was added to the mixture at −78 °C, and the reaction mixture was stirred for an additional 1 h. After this, the reaction was quenched with 5 M HCl, until the pH was 5–6, and the crude mixture was extracted with ethyl acetate (3 × 30 mL). The organic layers were then combined, dried over Na_2_SO_4_ and concentrated in vacuo. The crude product was purified using column chromatography with 3:7 EtOAc:hexane as an eluent to afford 3-((4-(benzyloxy)-3-methoxyphenyl)(hydroxy)methyl)-4-(3,4-dimethoxybenzyl)dihydrofuran-2(3H)-one (1.10 g, 2.30 mmol, 53%) as a yellow oil. ^1^H NMR (400 MHz, CDCl_3_): *δ*2.08 (*dd*, *J* = 13.8, 5.0 Hz, 1H), 2.17–2.21 (*m*, 1H), 2.25 (*dd*, *J* = 13.8, 7.1 Hz, 1H), 2.35–2.52 (*m*, 3H), 2.58–2.84 (*m*, 4H), 3.77 (*s*, 3H), 3.78 (*s*, 3H), 3.83 (*s*, 9H), 3.88–3.95 (*m*, 2H), 3.89 (*s*, 3H), 4.08–4.13 (*t*, *J* = 7.9 Hz, 1H), 4.29 (*dd*, *J* = 8.6, 8.2 Hz, 1H), 4.80 (*d*, *J* = 8.1 Hz, 1H), 5.14 (*s*, 2H), 5.15 (*s*, 2H), 5.26 (*d*, *J* = 3.0 Hz, 1H), 6.33–6.44 (*m*, 4H), 6.62–6.64 (*d*, *J* = 8.1 Hz, 1H), 6.68–6.70 (*d*, *J* = 8.1 Hz, 1H), 6.74 (*dd*, *J* = 8.3, 1.6 Hz, 1H), 6.81 (*dd*, *J* = 5.1, 3.1 Hz, 1H), 6.87 (*d*, *J* = 0.6 Hz, 2H), 6.99 (*s*, 1H), 7.27–7.46 (*m*, 10H); ^13^C NMR (100 MHz, CDCl_3_): *δ*36.4, 38.0, 39.1, 39.8, 51.5, 52.7, 55.7, 55.7, 55.8, 55.8, 55.9, 56.0, 70.9, 71.0, 71.6, 72.0, 72.6, 74.4, 108.9, 110.0, 111.1, 111.2, 111.6, 111.7, 113.7, 113.8, 117.3, 119.0, 120.3, 120.4, 2 × 127.1, 2 × 127.2, 2 × 127.9, 4 × 128.5, 130.3, 130.3, 133.17, 134.0, 134.7, 136.9, 147.5, 147.6, 147.7, 148.3, 148.8, 148.9, 149.7, 150.1, 178.3, 179.1; HRMS (ESI+, *m*/*z*): calculated for C_28_H_30_O_7_ [M+Na]^+^ 501.1884, found [M+Na]^+^ 501.1863.

##### Preparation of 4-(3,4-Dimethoxybenzyl)-3-(4-hydroxy-3-methoxybenzyl)dihydrofuran-2(3H)-one ((±)-TTPG-A)

A solution of 3-((4-(benzyloxy)-3-methoxyphenyl)(hydroxy)methyl)-4-(3,4-dimethoxybenzyl)dihydrofuran-2(3H)-one (424.7 mg, 0.89 mmol) in the solvent mixture system of THF:Acetic acid (1:1) (10 mL) was stirred at room temperature under hydrogen (H_2_) atmosphere in the presence of Pd(OH)_2_/C (12.46 mg, 0.18 mmol) overnight. After this, the metal catalyst was filtered out over a celite pad and the filtrate was then concentrated in vacuo. The crude product was purified using column chromatography with 3:7 EtOAc:hexane as an eluent to afford 4-(3,4-dimethoxybenzyl)-3-(4-hydroxy-3-methoxybenzyl)dihydrofuran-2(3H)-one (160.8 mg, 0.43 mmol, 49%) as a yellow oil. ^1^H NMR (400 MHz, CDCl_3_): *δ*2.47–2.66 (*m*, 4H), 2.86–2.96 (*m*, 2H), 3.81 (*d*, *J* = 2.6 Hz, 6H), 3.85 (*d*, *J* = 3.0 Hz, 3H), 3.86–3.91 (*m*, 1H), 4.09–4.18 (*m*, 1H), 5.53 (*d*, *J* = 2.3 Hz, 1H), 6.46 (*s*, 1H), 6.53–6.55 (*d*, *J* = 8.3 Hz, 1H), 6.58–6.62 (*m*, 1H), 6.63 (*s*, 1H), 6.74 (*dd*, *J* = 8.1, 2.9 Hz, 1H), 6.82 (*dd*, *J* = 7.9, 3.0 Hz, 1H); ^13^C NMR (100 MHz, CDCl_3_): *δ*34.5, 38.2, 40.9, 46.6, 55.8, 55.8, 55.9, 71.3, 111.2, 111.5, 111.7, 114.1, 120.6, 122.1, 129.5, 130.2, 144.5, 146.7, 147.8, 149.0, 178.8; HRMS (E/Z+, *m*/*z*): calculated for C_21_H_24_O_6_ [M+Na]^+^ 395.1465, found [M+Na]^+^ 395.1489.

#### 4.1.3. Synthesis (±)-TTPG-B

##### Preparation of 4-Butoxy-3-methoxybenzaldehyde

To a suspension of vanillin (1.06 g, 6.99 mmol) and potassium carbonate (K_2_CO_3_) (1.93 g, 13.97 mm) in 40 mL of anhydrous acetone, was added bromobutane (1.52 mL, 13.98 mmol) and KI (1.16 g, 6.99 mmol). The reaction mixture was then refluxed overnight. After this, the mixture was cooled down and filtered out to remove the K_2_CO_3_. The filtrate was then added to H_2_O (50 mL) and the mixture was extracted with ethyl acetate (3 × 50 mL). The organic layer was then combined, dried over Na_2_SO_4_, filtered and evaporated to afford 4-butoxy-3-methoxybenzaldehyde in 94% (1.36 g, 6.53 mmol) as a yellow oil. The product was used in the next step without further purification. ^1^H NMR (400 MHz, CDCl_3_): *δ*0.94–0.98 (*m*, 3H), 1.46–1.50 (*m*, 2H), 1.82–1.85 (*m*, 2H), 3.92 (*s*, 3H), 4.06–4.10 (*m*, 2H), 6.92–6.95 (*m*, 1H), 7.37–7.42 (*m*, 2H), 9.81 (*s*, 1H), ^13^C NMR (100 MHz, CDCl_3_): *δ*13.9, 19.2, 31.0, 59.1, 68.9, 109.3, 111.4, 126.9, 129.9, 149.9, 154.3, 191.1.

##### Preparation of 4-(4-Butoxy-3-methoxyphenyl)-3-(methoxycarbonyl)but-3-enoic Acid

Under N_2_ atmosphere, Na metal (5.87 g, 0.26 mol) was slowly added to anhydrous MeOH (200 mL), to generate sodium methoxide (NaOMe). After this, dimethyl succinate (8.7 mL, 69.3 mmol) was slowly added to the suspension of NaOMe, followed by stirring continuously at room temperature for 15 min. Then, the solution of 4-butoxy-3-methoxybenzaldehyde (12.88 g, 61.84 mmol) in dry MeOH (100 mL) was added to the previous suspension and then the mixture was allowed to reflux overnight. After completion, the reaction mixture was cooled down and then worked up with conc. HCl until the pH was 1–2. Next, the reaction mixture was evaporated to remove the MeOH. Then, H_2_O (100 mL) was added, and the mixture was extracted with ethyl acetate (3 × 100 mL). The organic layers were combined, dried over Na_2_SO_4_, and concentrated in vacuo. The crude product was purified using column chromatography with 30–50% EtOAc/hexane as an eluent to afford 4-(4-butoxy-3-methoxyphenyl)-3-(methoxycarbonyl)but-3-enoic acid (16.59 g, 51.46 mmol, 85% yield) as a yellow solid. ^1^H NMR (400 MHz, CDCl_3_): *δ*0.98 (*t*, *J* = 7.4 Hz, 3H), 1.47–1.53 (*m*, 2H), 1.82–1.85 (*m*, 2H), 3.70 (*s*, 2H), 3.85 (*s*, 3H), 3.87 (*s*, 3H), 4.05 (*t*, *J* = 6.8 Hz, 2H), 6.90 (*d*, *J* = 7.32 Hz, 1H), 6.97–6.99 (*m*, 2H), 7.86 (*s*, 1H); ^13^C NMR (100 MHz, CDCl_3_): *δ*13.8, 19.1, 31.2, 35.5, 52.3, 56.0, 69.6, 113.8, 114.6, 125.4, 125.9, 129.2, 140.6, 149.7, 150.2, 168.4, 170.0; HRMS (E/Z+, *m*/*z*): calculated for C_17_H_22_O_6_ [M+Na]^+^ 345.1308, found [M+Na]^+^ 345.1301.

##### Preparation of 3-(3,4-Dimethoxybenzyl)-4-methoxy-4-oxobutanoic Acid

A suspension of 4-(4-butoxy-3-methoxyphenyl)-3-(methoxycarbonyl)but-3-enoic acid (16.59 g, 51.46 mmol) and palladium on charcoal (Pd/C) (2.61 g, 0.03 mol) in MeOH (100 mL) was stirred at room temperature under hydrogen (H_2_) atmosphere overnight. After this, the reaction mixture was filtered over a celite pad and washed with MeOH twice. The filtrate was concentrated in vacuo to provide 3-(3,4-dimethoxybenzyl)-4-methoxy-4-oxobutanoic acid (14.99 g, 46.37 mmol, 91% yield) as a yellow oil without further purification. ^1^H NMR (400 MHz, CDCl_3_): *δ*0.97 (*t*, *J* = 2.9 Hz, 3H), 1.45–1.51 (*m*, 2H), 1.79–1.82 (*m*, 2H), 2.45 (*dd*, *J* = 17.1 Hz, 4.6 Hz, 1H), 2.67–2.74 (*m*, 2H), 2.97–3.07 (*m*, 2H), 3.67 (*s*, 3H), 3.84 (*s*, 3H), 3.99 (*t*, *J* = 6.8 Hz, 2H), 6.64–6.67(*m*, 2H), 6.78 (*d*, *J* = 8.7 Hz, 1H); ^13^C NMR (100 MHz, CDCl_3_): *δ*14.0, 19.3, 29.8, 31.3, 34.7, 37.4, 43.1, 52.1, 56.1, 68.8, 112.6, 113.0, 130.4, 147.6, 149.5, 174.8, 177.1; HRMS (E/Z+, *m*/*z*): calculated for C_17_H_24_O_6_ [M+Na]^+^ 347.1465, found [M+Na]^+^ 347.1498.

##### Preparation of 4-(4-Butoxy-3-methoxybenzyl)dihydro-furan-2(3H)-one

A solution of 3-(3,4-dimethoxybenzyl)-4-methoxy-4-oxobutanoic acid (14.99 g, 46.37 mmol) in MeOH (200 mL) was stirred at room temperature in the presence of KOH (2.61 g, 46.64 mmol) for 15 min. After this, the reaction mixture was evaporated to dryness. Next, EtOH (200 mL) was added, followed by CaCl_2_ (5.46, 49.16 mmol) and the mixture was stirred continuously for 15 min. Then, the mixture of KOH (1.04 g, 18.56 mmol) and NaBH_4_ (3.84 g, 0.11 mol) in EtOH (50 mL) was slowly added to the reaction mixture at 0 °C, followed by stirring at room temperature for 3 h. After completion, the reaction mixture was quenched with conc. HCl until the pH was 4–5, and then filtered over a celite pad, to remove the solid. H_2_O (100 mL) was added to the filtrate and the mixture was extracted with ethyl acetate (3 × 200 mL). The organic layers were combined, dried over Na_2_SO_4_, filtered, and concentrated in vacuo to generate 4-(4-butoxy-3-methoxybenzyl)dihydrofuran-2(3H)-one (10.59 g, 38.05 mmol, 82% yield) without any further purification. ^1^H NMR (400 MHz, CDCl_3_): *δ*0.95 (*t*, *J* = 2.8 Hz, 3H), 1.43–1.49 (*m*, 2H), 1.75–1.82 (*m*, 2H), 2.23–2.29 (*m*, 1H), 2.54–2.61 (*m*, 1H), 2.66–2.69 (*m*, 2H), 2.78–2.82 (*m*, 1H), 3.82 (*s*, 3H), 3.95–4.03 (*m*, 3H), 4.28–4.32 (*m*, 3H), 6.63–6.65 (*m*, 2H), 6.79 (*d*, *J* = 7.0 Hz, 1H); ^13^C NMR (100 MHz, CDCl_3_): *δ*14.0, 19.3, 31.3, 34.3, 37.4, 38.7, 56.2, 68.9, 72.8, 112.4, 113.2, 120.8, 130.8, 147.6, 149.6, 177.1; HRMS (E/Z+, *m*/*z*): calculated for C_16_H_22_O_4_ [M+Na]^+^ 301.1410, found [M+Na]^+^ 301.1410.

##### Preparation of 3-((4-(Benzyloxy)-3-methoxyphenyl)(hydroxy)methyl)-4-(4-butoxy-3-methoxybenzyl)dihydrofuran-2(3H)-one

Under N_2_ atmosphere, 2M lithium diisopropylamide (LDA) (3.6 mL, 7.20 mmol) was slowly added to the mixture of 4-(4-butoxy-3-methoxybenzyl)dihydrofuran-2(3H)-one (1.01 g, 3.63 mmol) in dry THF (5 mL) at −78 °C, and the reaction mixture was allowed to stir for 1 h. 4-(Benzyloxy)-3-methoxybenzaldehyde (1.70 g, 7.51 mmol) in dry THF (5 mL) was added to the mixture at −78 °C, and the reaction mixture was stirred for an additional 1 h. After this, the reaction was quenched with 5M HCl until the pH reached 5–6, and the crude mixture was extracted with ethyl acetate (3 × 20 mL). All of the organic layers were combined, dried over anhydrous Na_2_SO_4_ and concentrated in vacuo. The crude product was purified using column chromatography with 3:7 EtOAc:hexane as an eluent to afford 3-((4-(benzyloxy)-3-methoxyphenyl)(hydroxy)methyl)-4-(4-butoxy-3-methoxybenzyl) dihydrofuran-2(3H)-one (0.85 g, 1.63 mmol, 45%) as a yellow oil. ^1^H NMR (400 MHz, CDCl_3_): *δ*0.94–0.98 (*td*, *J* = 7.4, 1.7 Hz, 6H), 1.43–1.45 (*m*, 4H), 1.76–1.83 (*m*, 4H), 2.07 (*dd*, *J* = 13.8, 5.0 Hz, 2H), 2.13–2.19 (*m*, 2H), 2.26 (*dd*, *J* = 13.8, 6.9 Hz, 1H), 2.36–2.49 (*m*, 2H), 2.58–2.67 (*m*, 2H), 2.76–2.84 (*dt*, *J* = 14.8, 7.5 Hz, 1H), 3.75 (*s*, 3H), 3.77 (*s*, 3H), 3.84 (*s*, 3H), 3.89–3.91 (*m*, 1H), 3.90 (*s*, 3H), 3.92–3.99 (*m*, 5H), 4.10–4.14 (*m*, 1H), 4.28 (*t*, *J* = 8.4 Hz, 1H), 4.80 (*d*, *J* = 8.2 Hz, 1H), 5.14–5.15 (*m*, 4H), 5.27 (*d*, *J* = 3.1 Hz, 1H), 6.33–6.39 (*m*, 4H), 6.68 (*dd*, *J* = 16.8, 8.0 Hz, 2H), 6.74 (*dd*, *J* = 8.2, 1.9 Hz, 1H), 6.83 (*dd*, *J* = 6.4, 5.2 Hz, 2H), 6.88 (*s*, 1H), 6.88 (*s*, 1H), 7.00 (*s*, 1H), 7.27–7.44 (*m*, 10H); ^13^C NMR (100 MHz, CDCl_3_): *δ*13.8, 13.8, 19.2, 19.2, 31.2, 31.3, 36.5, 38.0, 39.0, 39.9, 51.5, 52.7, 55.9, 56.0, 56.1, 68.7, 68.7, 71.0, 71.0, 71.7, 72.1, 72.5, 74.5, 109.0, 110.0, 112.2, 112.2, 112.8, 113.0, 113.8, 113.9, 117.4, 119.0, 120.4, 120.5, 2 × 127.2, 2 × 127.3, 2 × 127.9, 4 × 128.5, 130.2, 130.2, 133.2, 133.9, 136.8, 136.9, 147.3, 147.4, 147.6, 148.3, 149.3, 149.4, 149.8, 150.2, 178.3, 179.1; HRMS (E/Z+, *m*/*z*): calculated for C_31_H_36_O_7_ [M+Na]^+^ 543.2353, found [M+Na]^+^ 543.2352.

##### Preparation of 4-(4-Butoxy-3-methoxybenzyl)-3-(4-hydroxy-3-methoxybenzyl)dihydrofuran-2(3H)-one ((±)-TTPG-B)

A solution of 3-((4-(benzyloxy)-3-methoxyphenyl)(hydroxy)methyl)-4-(4-butoxy-3-methoxybenzyl)dihydrofuran-2(3H)-one (159.9 mg, 0.31 mmol) in the solvent mixture system of THF:Acetic acid (1:1) (4 mL) was stirred at room temperature under hydrogen (H_2_) atmosphere in the presence of Pd(OH)_2_/C (4.61 mg, 0.066 mmol), overnight. After this, the metal catalyst was filtered out over a Celite pad and the filtrate was then concentrated in vacuo. The crude product was purified using column chromatography with 3:7 EtOAc:hexane as an eluent to afford 4-(4-butoxy-3-methoxybenzyl)-3-(4-hydroxy-3-methoxybenzyl)dihydrofuran-2(3H)-one (60.39 mg, 0.15 mmol, 47%) as a yellow oil. ^1^H NMR (400 MHz, CDCl_3_) *δ*0.95–0.99 (*t*, *J* = 7.4 Hz, 3H), 1.48 (*dd*, *J* = 15.1, 7.5 Hz, 2H), 1.81 (*dt*, *J* = 14.7, 6.9Hz, 2H), 2.45–2.66 (*m*, 4H), 2.91–2.95 (*m*, 2H), 3.79 (*s*, 3H), 3.82 (*s*, 3H), 3.85–3.89 (*m*, 1H), 3.97 (*t*, *J* = 6.8 Hz, 2H), 4.12 (*dd*, *J* = 9.1, 7.1 Hz, 1H), 6.47 (*d*, *J* = 1.9 Hz, 1H), 6.52 (*dd*, *J* = 8.1, 1.9 Hz, 1H), 6.61 (*dd*, *J* = 8.0, 1.9 Hz, 1H), 6.65 (*d*, *J* = 1.8 Hz, 1H), 6.75 (*d*, *J* = 8.1 Hz, 1H), 6.82 (*d*, *J* = 8.0 Hz, 1H); ^13^C NMR (100 MHz, CDCl_3_): *δ*13.8, 19.2, 31.2, 34.4, 38.1, 40.9, 46.6, 55.8, 55.9, 68.7, 71.3, 111.5, 112.3, 113.0, 114.1, 120.6, 122.1, 129.5, 130.4, 144.5, 146.7, 147.4, 149.4, 178.8; HRMS (E/Z+, *m*/*z*): calculated for C_24_H_30_O_7_ [M+Na]^+^ 437.1935, found [M+Na]^+^ 437.1934. ^1^H, ^13^C NMR and HRMS spectrum of compounds (See Supplementary Materials).

### 4.2. Cell Culture

Human-breast adenocarcinoma (MCF-7) (ATCC^®®^) HTB-22), MDA-MB-213 (ATCC^®®^HTB-26), MDA-MB-468 (ATCC^®®^ HTB-132)) cells were purchased from the American Type Culture Collection (ATCC) (Manassas, VA, USA). Human-colon adenocarcinoma (HT-29) cells were supplied from Assoc. Prof. Dr. Ruedeekorn Wiwattanapatapee (Department of Pharmaceutical Technology, Faculty of Pharmaceutical Sciences, Prince of Songkla University (PSU), Songkhla, Thailand). Cholangiocarcinoma (KKU-M213 and KKU-K100) cells were contributed by Asst. Prof. Dr. Mutita Jungking (Division of Molecular Medicine, Department of Research and Development, Faculty of Medicine Siriraj Hospital, Mahidol University, Bangkok, Thailand). Ovarian cancer (A2780) cells were purchased from AddexBio (San Diego, CA, USA).

For normal cells, monkey kidney epithetical (Vero, ATCC^®®^ CCL-81) cells were purchased from ATCC^®®^ (Manassas, VA, USA). Mouse fibroblast cells (L-929) were donated by Assoc. Prof. Dr. Jasadee Kaewsrichan (Drug Delivery System Excellence Center, Faculty of Pharmaceutical Sciences, PSU, Songkhla, Thailand). Immortal cholangiocyte (MMNK-1) cells were obtained from Asst. Prof. Dr. Mutita jungking.

MCF-7 and A2780 cells were cultured in Roswell Park Memorial Institute-1640 (RPMI-1640) culture medium. MDA-MB-213, MDA-MB-468, HT-29, KKU-M213, KKU-K100, MMNK-1, Vero and L-929 cells were grown in Dulbecco’s modified Eagle medium (DMEM). RPMI-1640 and DMEM were supplemented with 10% FBS (GIBCO BRL) with 2 mmol/L of L-glutamine (Invitrogen) and an antibiotic mixture (Invitrogen) of 100 units/mL of penicillin and 100 μg/mL of streptomycin. All cells were maintained by incubating in a 5% CO_2_ atmosphere, at 37 °C, and 95% relative humidity [40].

### 4.3. Cytotoxicity Assay

To evaluate the cytotoxicity effect of (±)-kusunokinin derivatives, the 3-(4,5-dimethyl thiazol-2yl)-2,5-diphenyltetrazolium bromide (tetrazolium salt MTT, Cat No: M6494, Invitrogen, MA, USA) colorimetric assay was performed. Cells were seeded into 96-well plates. MCF-7, MDA-MB-213, MDA-MB-468 and HT-29 cells were seeded at a density of 2 × 10^4^ cells/well. Vero, L-929 and A2780 cells were seeded at a density of 1 × 10^4^ cells/well. KKU-M213 cells were seeded at a density of 7.5 × 10^3^ cells/well. All seeded cells were incubated for at least 24 h for adhering. The complete medium was mixed with compounds at different concentrations (0.625, 1.25, 2.5, 5.0 and 10 µg/mL) and incubated for 72 h. After incubation, live cells were detected as previously described. The absorbance was measured at 570 and 650 nM in the microplate reader Varioskan™ LUX Multimode Microplate Reader (Thermo Fisher Scientific, MA, USA). Each condition of treatment was analyzed individually 3 times. The half-maximal inhibitory concentration (IC_50_) values were determined and calculated, as previously described [40].

### 4.4. Cell Cycle Analysis Assay

This assay was performed using Muse^®®^ kit and carried out using the Muse^®®^ cell analyzer, according to the manufacturer’s protocol. Briefly, KKU-M213 cells were seeded in 24-well plates at a density of 1 × 10^5^ cells/well. After incubation for 24 h, the medium was removed and replaced with a fresh culture-medium either with or without an IC_50_ concentration of (±)-kusunokinin, (±)-TTPG-A or (±)-TTPG-B. Then, the cells were harvested at 24, 48 and 72 h, and resuspended in 1X PBS buffer. The cells were stained with propidium iodide (PI) (Millipore’s Muse^®®^ Cell Cycle Kit (Catalog No. MCH100106, Merck Millipore, Darmstadt, Germany). The percentages of the cells in G0/G1, S, and G2/M phases of the cell cycle were analyzed using the MUSE^®®^ Cell Analyzer (Merck Millipore, Darmstadt, Germany).

### 4.5. Apoptosis Assay

The apoptotic cells were detected using the Muse^®®^ Annexin V Dead Cell Kit (Catalog No. MCH100105, Merck Millipore). In this study, KKU-M213 cells (1 × 10^4^ cells/well) were seeded into 24-well plates. Cells were treated with an IC_50_ concentration of (±)-kusunokinin, (±)-TTPG-A and (±)-TTPG-B at 4.47, 0.07 and 0.01 µM, respectively, for 72 h. Non-treated cells served as control. In addition, cells were incubated with 0.15 µM of each compound for 48 h. The treated cells were harvested and cell pellets were incubated in 100 μL of Muse^®®^ FITC-Annexin V apoptosis staining kit and 100 μL of PI for 30 min. The signal fluorescence was observed with flow cytometry, using a Muse^®®^ Cell Analyzer (Merck Millipore, Darmstadt, Germany).

### 4.6. Multi-Caspase Activity Assay

The percentage of cells with multi-caspase activity was determined using a Muse^®®^ multi-caspase assay kit (Catalog No. MCH100109, Merck Millipore), following the manufacturer’s instructions. In this assay, the experiment was carried out in the same way as the apoptosis assay. After treatment, the treated cells were harvested in 1X caspase buffer. The Muse™ multicaspase reagent and Muse™ caspase 7-aminoactinomycin D (7-AAD) working solution were stained in each sample. The multi-caspase activity was performed following the protocol provided by the manufacturer, and analyzed using the MUSE^®®^ Cell Analyzer [19].

### 4.7. Statistical Analysis

All data were analyzed using Student’s *t*-test on Microsoft Excel, and represented as the mean ± standard deviation (SD). A *p-*value less than *˂*0.05 was considered statistically significant.

## 5. Conclusions

In conclusion, these findings reveal that (±)-TTPG-A and (±)-TTPG-B exhibited the strongest cytotoxic effect on cholangiocarcinoma cells, especially KKU-M213 cells. Both compounds suppressed cell-cycle progression at the S and G0/G1 phases, respectively. (±)-TTPG-A and (±)-TTPG-B induced apoptosis, and modulated the activity of several caspases.

## Figures and Tables

**Table 1:** The half-maximal-inhibitory-concentration (IC_50_) values of (±)-TTPG-A and (±)-TTPG-B for inhibition of viability of cancer and normal cells.

Cells	IC_50_ (µM)		
	(±)-Kusunokinin	(±)-TTPG-A	(±)-TTPG-B
Breast cancer MCF-7	4.23 ± 0.21	3.47 ± 0.02 *	5.94 ± 0.04
MDA-MB-468	5.19 ± 0.02	6.38 ± 0.04	0.43 ± 0.01 *
MDA-MB-231	9.23 ± 0.12	6.50 ± 0.01 *	1.83 ± 0.04 *
Cholangiocarcinoma KKU-M213	4.47 ± 0.04	0.07 ± 0.01 *	0.01 ± 0.001 *
KKU-K100	4.46 ± 0.29	0.76 ± 0.05 *	1.53 ± 0.01 *
Colon cancer HT-29	5.34 ± 0.03	Not inhibited	Not inhibited
Ovarian cancer A2780	4.52 ± 0.03	6.13 ± 0.04	0.05 ± 0.01 *
Normal cells MMNK-1 Vero L929	6.79 ± 0.04 Not inhibited 9.75 ± 0.39	6.30 ± 0.01 14.53 ± 0.02 Not inhibited	1.53 ± 0.01 15.15 ± 0.16 Not inhibited

Not inhibited: IC_50_ not observed at the maximum concentration at 27 µM. *: *p* < 0.05, compared with (±)-kusunokinin.

## Data Availability

All data represented in this study are available in article on reasonable request, from the corresponding author.

## Supplementary Materials

The following supporting information can be downloaded at: https://www.mdpi.com/article/10.3390/molecules27238291/s1, ^1^H, ^13^C NMR and HRMS spectrum of compounds.

## Sample Availability

Samples of the compounds are not available from the authors.
