# Supplementary material for: Anticancer Activity of (±)-Kusunokinin Derivatives towards Cholangiocarcinoma Cells
Source: Molecules. 2022 Nov 28;27(23):8291. doi: 10.3390/molecules27238291 (PMC9735782; doi:10.3390/molecules27238291)

^{1}H NMR spectrum of 4-(3,4-dimethoxybenzyl)-3-(4-hydroxy-3-methoxybenzyl)dihydro furan-2(3H)-one: (±)-TTPG-A

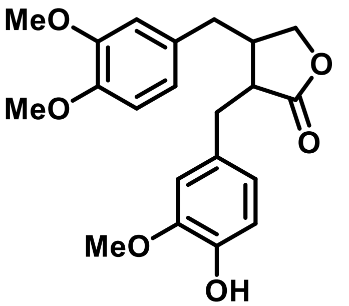

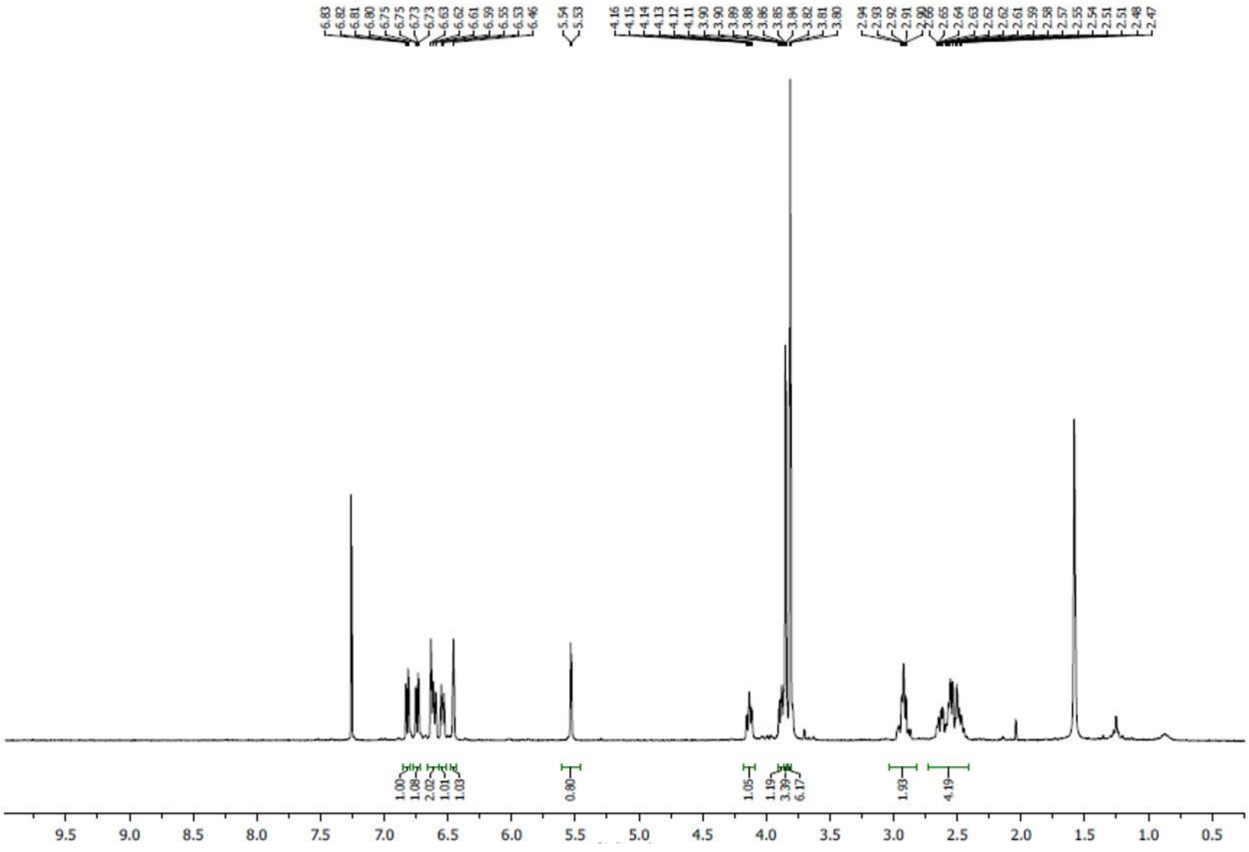

^{13}C NMR spectrum of 4-(3,4-dimethoxybenzyl)-3-(4-hydroxy-3-methoxybenzyl)dihydro furan-2(3H)-one: (±)-TTPG-A

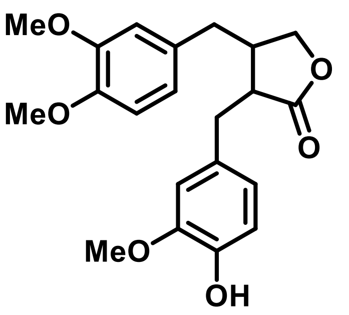

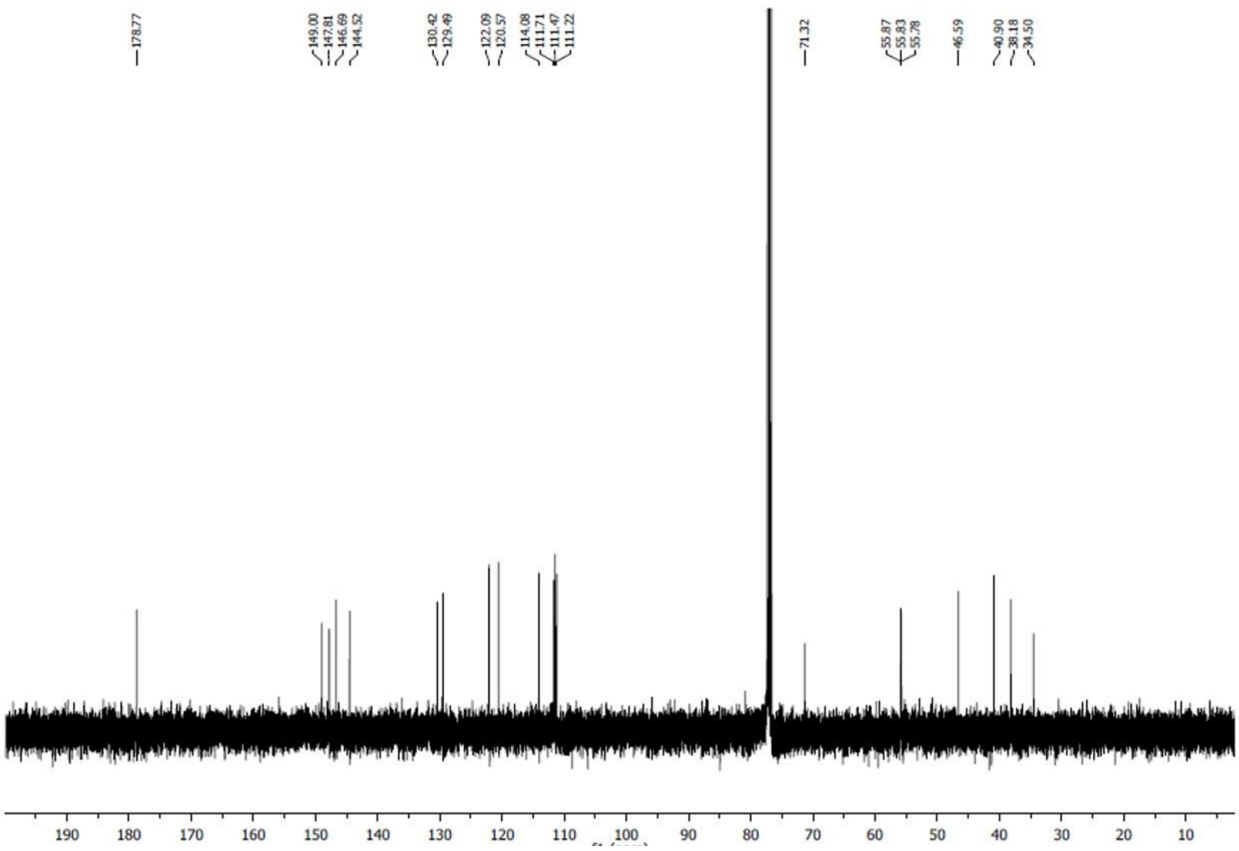

HRMS spectrum of 4-(3,4-dimethoxybenzyl)-3-(4-hydroxy-3-methoxybenzyl)dihydro furan-2(3H)-one: (+)-TTPG-A

Analysis Name	\\10.222.72.169\Data\chawanee\OMe_OMe_OH_OMe_hydro re.d				
Method	NaFormate_pos low.m	Operator	Sutichai	Ext: 3560
Sample Name	OMe_OMe_OH_OMe_hydro re	Instrument	micrOTOF	Bruker
		Calibrate by	Sodium Formate	

Acquisition Parameter

Source Type	ESI	Ion Polarity	Positive	Set Nebulizer	0.4 Bar
Focus	Not active			Set Dry Heater	180 □C
Scan Begin	50 m/z	Set Capillary	4500 V	Set Dry Gas	5.0 l/min
Scan End	3000 m/z	Set End Plate Offset	-500 V	Set Divert Valve	Source

Spectrum View

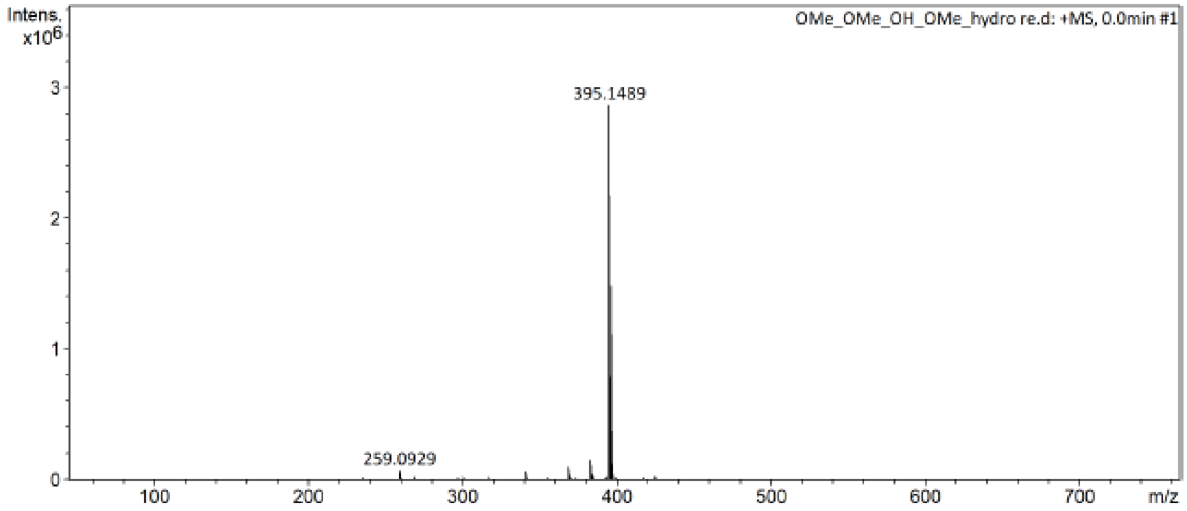

Compound Spectra

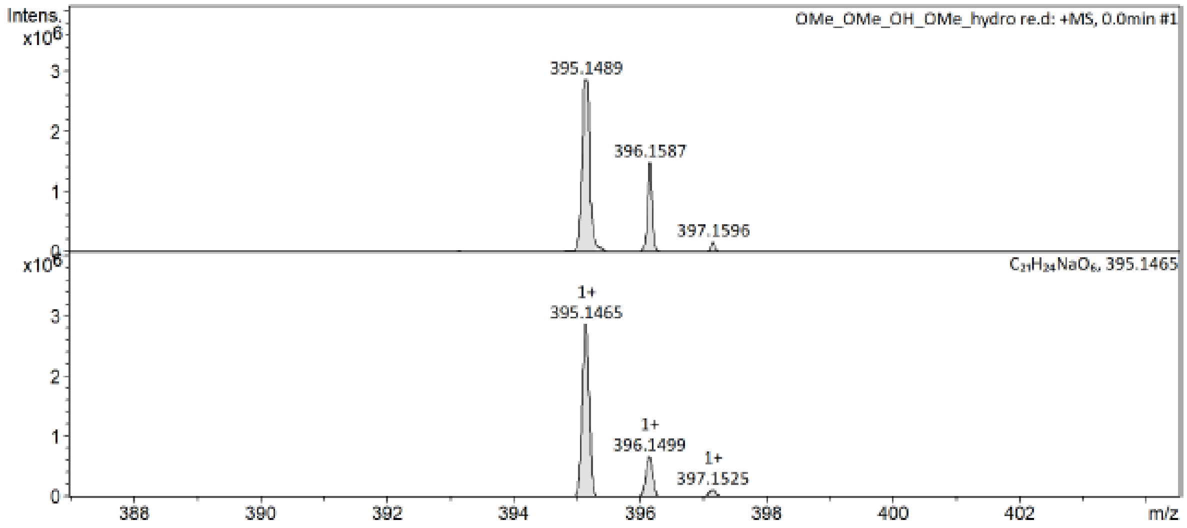

^{1}H NMR spectrum of 4-(4-butoxy-3-methoxybenzyl)-3-(4-hydroxy-3-methoxybenzyl) dihydrofuran-2(3H)-one: (±)-TTPG-B

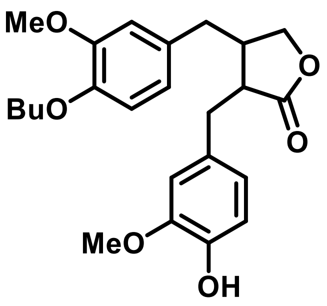

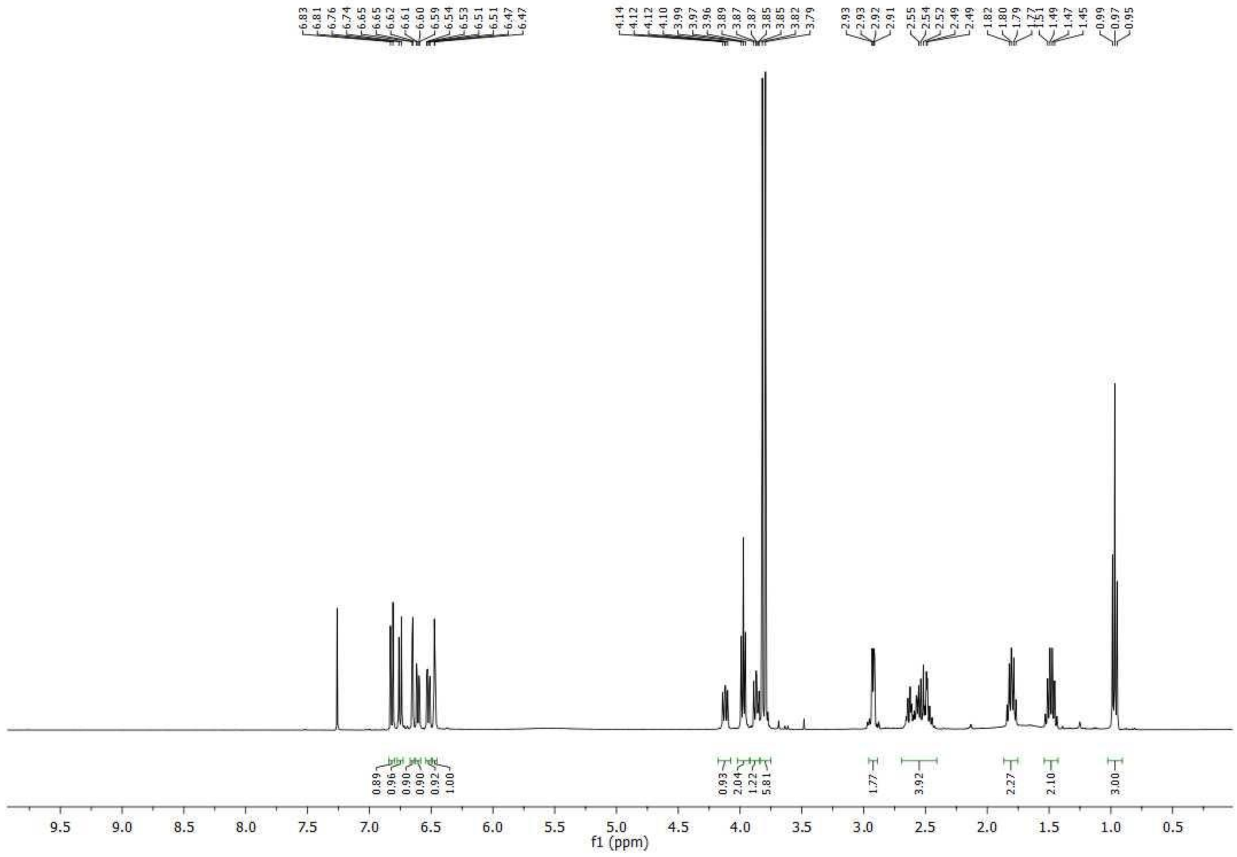

^{13}C NMR spectrum of 4-(4-butoxy-3-methoxybenzyl)-3-(4-hydroxy-3-methoxybenzyl) dihydrofuran-2(3H)-one: (±)-TTPG-B

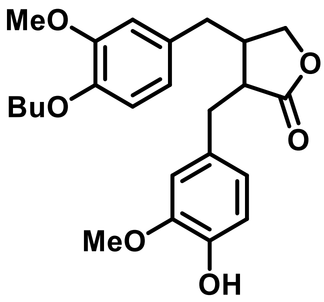

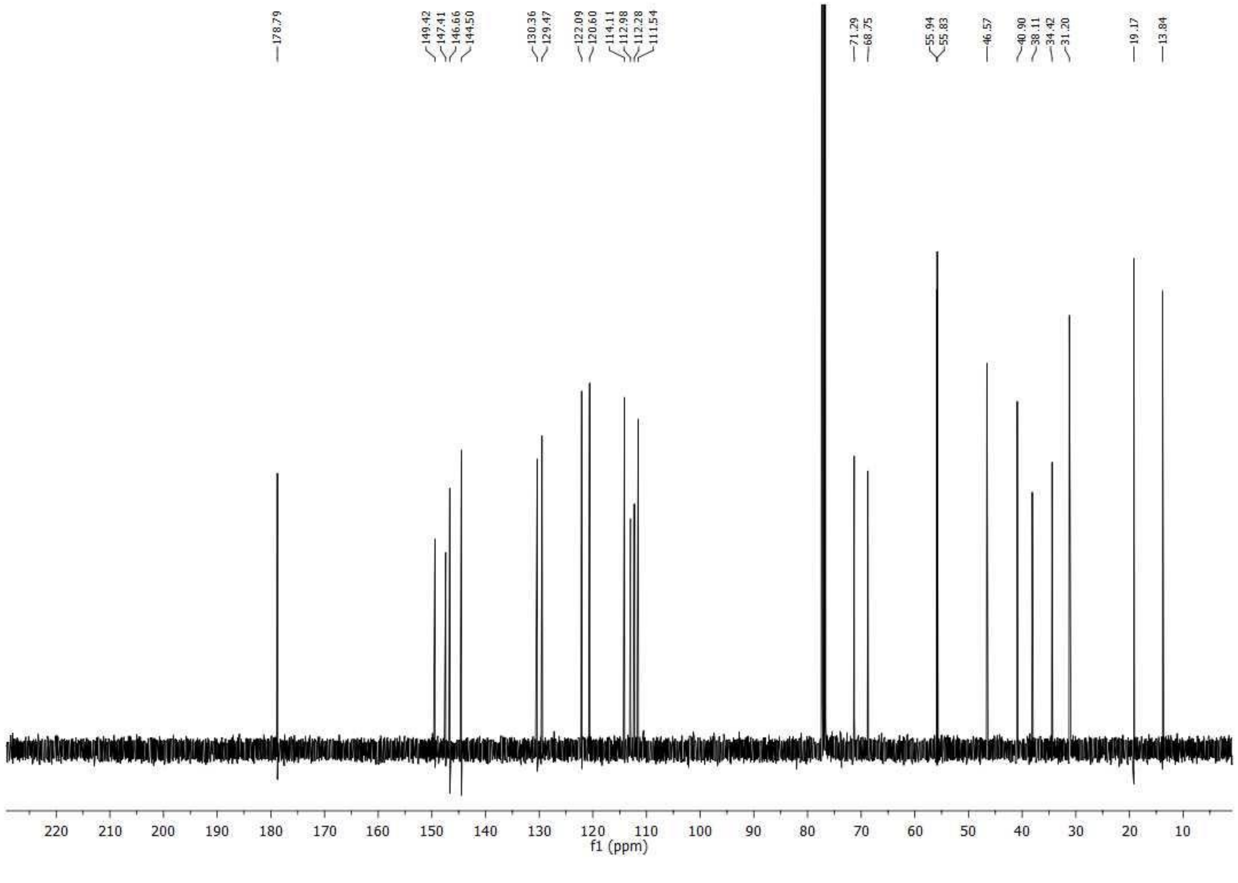

HRMS spectrum of 4-(4-butoxy-3-methoxybenzyl)-3-(4-hydroxy-3-methoxybenzyl) dihydrofuran-2(3H)-one: (+)-TTPG-B

Analysis Name	\\10.222.72.169\Data\chawanee\OMe OBu OMe OH final hydro re.d				
Method	NaFormate_pos low.m	Operator	Sutichai	Ext: 3560	
Sample Name	OMe OBu OMe OH final hydro re	Instrument	micrOTOF	Bruker	
		Calibrate by	Sodium Formate		

Acquisition Parameter

Source Type	ESI	Ion Polarity	Positive	Set Nebulizer	0.4 Bar
Focus	Not active			Set Dry Heater	180 □C
Scan Begin	50 m/z	Set Capillary	4500 V	Set Dry Gas	5.0 l/min
Scan End	3000 m/z	Set End Plate Offset	-500 V	Set Divert Valve	Source

Spectrum View

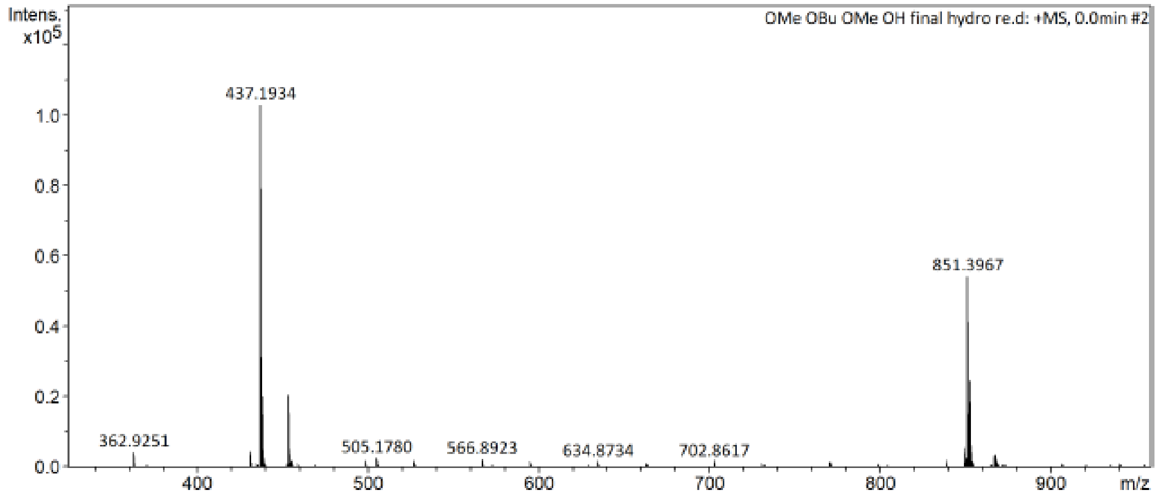

Compound Spectra

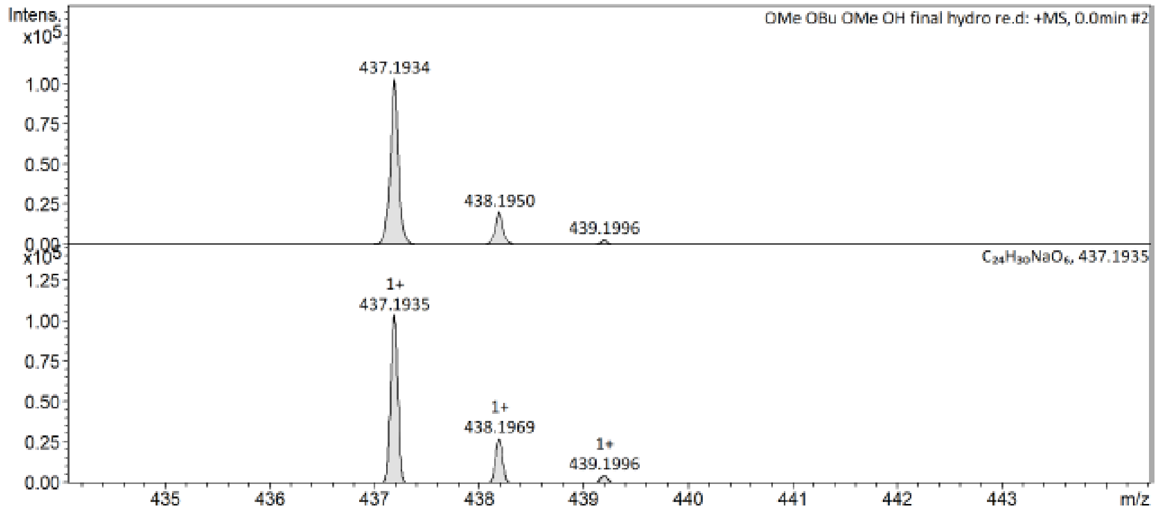

Supplement: Supplementary file 1 [file molecules-27-08291-s001.zip › molecules-2009442-supplementary.pdf]
